# Comprehensive Analysis and Reinforcement Learning of Hypoxic Genes Based on Four Machine Learning Algorithms for Estimating the Immune Landscape, Clinical Outcomes, and Therapeutic Implications in Patients With Lung Adenocarcinoma

**DOI:** 10.3389/fimmu.2022.906889

**Published:** 2022-06-10

**Authors:** Zhaoyang Sun, Yu Zeng, Ting Yuan, Xiaoying Chen, Hua Wang, Xiaowei Ma

**Affiliations:** ^1^ Department of Laboratory Medicine, Ren Ji Hospital, Shanghai Jiao Tong University School of Medicine, Shanghai, China; ^2^ Department of Thyroid and Neck Tumor, Tianjin Medical University Cancer Institute and Hospital, National Clinical Research Center for Cancer, Key Laboratory of Cancer Prevention and Therapy, Tianjin’s Clinical Research Center for Cancer, Tianjin, China; ^3^ Institute of Molecular Medicine, Department of Laboratory Medicine, Shanghai Key Laboratory for Nucleic Acid Chemistry and Nanomedicine, State Key Laboratory of Oncogene and Related Genes, Ren Ji Hospital, Shanghai Jiao Tong University School of Medicine, Shanghai, China

**Keywords:** lung adenocarcinoma, hypoxia gene, immune landscape, overall survival, prognosis, therapeutic implications

## Abstract

**Background:**

Patients with lung adenocarcinoma (LUAD) exhibit significant heterogeneity in therapeutic responses and overall survival (OS). In recent years, accumulating research has uncovered the critical roles of hypoxia in a variety of solid tumors, but its role in LUAD is not currently fully elucidated. This study aims to discover novel insights into the mechanistic and therapeutic implications of the hypoxia genes in LUAD cancers by exploring the potential association between hypoxia and LUAD.

**Methods:**

Four machine learning approaches were implemented to screen out potential hypoxia-related genes for the prognosis of LUAD based on gene expression profile of LUAD samples obtained from The Cancer Genome Atlas (TCGA), then validated by six cohorts of validation datasets. The risk score derived from the hypoxia-related genes was proven to be an independent factor by using the univariate and multivariate Cox regression analyses and Kaplan–Meier survival analyses. Hypoxia-related mechanisms based on tumor mutational burden (TMB), the immune activity, and therapeutic value were also performed to adequately dig deeper into the clinical value of hypoxia-related genes. Finally, the expression level of hypoxia genes was validated at protein level and clinical samples from LUAD patients at transcript levels.

**Results:**

All patients in TCGA and GEO-LUAD group were distinctly stratified into low- and high-risk groups based on the risk score. Survival analyses demonstrated that our risk score could serve as a powerful and independent risk factor for OS, and the nomogram also exhibited high accuracy. LUAD patients in high-risk group presented worse OS, lower TMB, and lower immune activity. We found that the model is highly sensitive to immune features. Moreover, we revealed that the hypoxia-related genes had potential therapeutic value for LUAD patients based on the drug sensitivity and chemotherapeutic response prediction. The protein and gene expression levels of 10 selected hypoxia gene also showed significant difference between LUAD tumors tissues and normal tissues. The validation experiment showed that the gene transcript levels of most of their genes were consistent with the levels of their translated proteins.

**Conclusions:**

Our study might contribute to the optimization of risk stratification for survival and personalized management of LUAD patients by using the hypoxia genes, which will provide a valuable resource that will guide both mechanistic and therapeutic implications of the hypoxia genes in LUAD cancers.

## Introduction

Lung cancer histology is determined according to the WHO classification based primarily on the light microscopic appearance of the malignant cells (adenocarcinoma, squamous carcinoma, large cell carcinomas, and small cell carcinoma) (1). Among lung cancers, lung adenocarcinoma (LUAD) is the most commonly diagnosed subtype, accounts for 40% of all diagnosed lung cancers, and has an average 5-year survival rate of only 15% (2, 3). The incidence of LUAD has increased significantly over the past two decades, especially among women (4). As a highly aggressive disease with significant heterogeneous prognosis across individuals, the molecular mechanisms underlying LUAD progression remain elusive (5). The International Union Against Cancer (UICC) tumor–node–metastasis (TNM) staging system was widely used for LUAD prognosis assessment (6). However, TNM-based clinical assessment method has so far proved inadequate in predicting clinical outcomes and treatment decision. Therefore, it has become one of the hot spots in clinical research to find more valuable prognosis indexes of LUAD.

Hypoxia, or lack of oxygen, is a feature of most solid tumors (7). Studies have shown that hypoxia-inducible factors (HIFs) are highly expressed in osteosarcoma stem cells (OSCs), and a significant decrease in stem cell proliferation and migratory activity was found after selective inhibition of HIF-1α or HIF-2α (8). During tumor progression, hypoxia develops when tumor growth exceeds the ability of available vasculature to supply tumor cells with oxygen and nutrients (9). Tumor hypoxia is one of the worst prognosis factors for survival (10). Multiple studies have demonstrated that hypoxia condition is an important cause of promoting the proliferation and angiogenesis, chemoradiotherapy resistance of cancer cells, migration, invasion, and metastatic growth at distant sites, which are significant obstacles to treatment and cause significant adverse prognostic ramifications (11–13). In LUAD, the upregulation of multiple hypoxic-related genes has been reported to have a significant prognostic value, such as *HIF-1α* (14), *NLUCAT1* (15), *TRB3* (16), *GBE*1 (17), and *CCL28* (18), highlighting the potential therapeutic value of targeting hypoxic-related genes, and the prognostic assessment and treatment decision. In view of the crucial role of hypoxic in the LUAD, hypoxic-related genes may be an effective way to predict the prognosis and therapeutic benefit for LUAD patients, individually.

**Figure 1:**
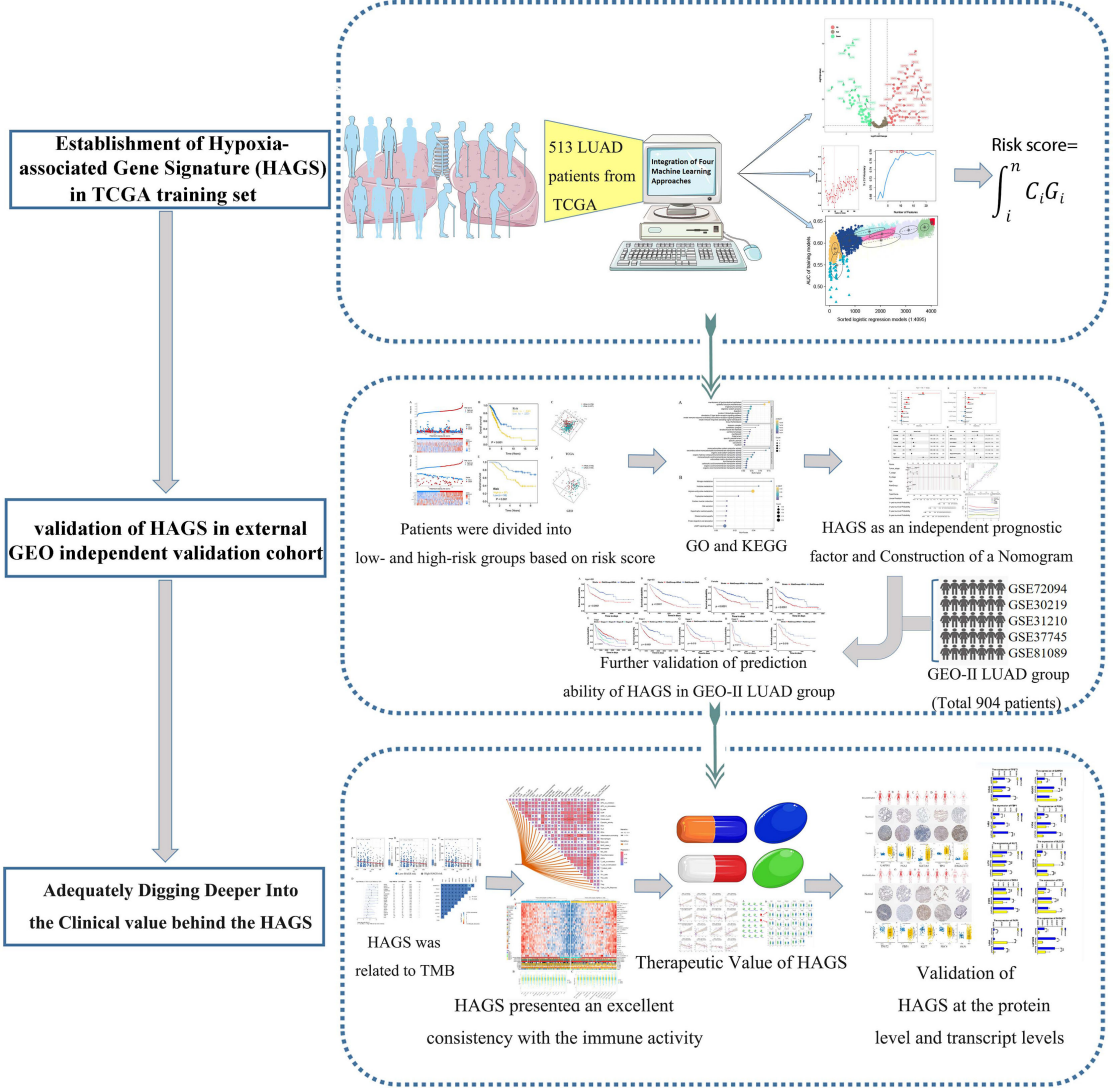
Flowchart for developing an individualized hypoxia-associated gene-based prognostic signature for LUAD.

In the present study, a range of machine learning and bioinformatic approaches were combined and used to excavate and screen robust candidate genes to explore in depth the potential correlation between hypoxia and LUAD, followed by the establishment and verification of an individualized hypoxia-derived signatures (**Figure 1**). Our findings provide further insight into the role of hypoxic-related genes in LUAD and provide a comprehensive demonstration that they are promising prognostic markers and therapeutic targets for LUAD.

## Methods

### Patient Samples

In March 2022, three LUAD tissues and their paired non-tumorous lung tissues were collected for quantitative real-time PCR (qRT-PCR) detection from the Ren Ji Hospital. All specimens were evaluated for histological features by pathologists according to criteria. The investigators obtained approval from the Ethics Committee of the Ren Ji Hospital, affiliated Shanghai Jiao Tong University School of Medicine to conduct the study (Ethics Approval Number KY2021-220-B). All procedures were carried out in accordance with the Declaration of Helsinki and relevant Chinese policies.

### RNA Isolation and Quantitative Real-Time PCR

Trizol reagent (Spark Jade, Qingdao China) was used to extract the nucleic acids from three pairs of LUAD tissues and their paired normal tissues according to the manufacturer’s instructions. Then, qRT-PCR reactions were performed with 2xHQ SYBR qPCR mix (ZOMANBIO, Beijing, China) by using the 7500 fast real-time PCR system ((Applied Biosystems, USA). The primers of 10 selected hypoxia genes in this study are outlined in **Table 1**.

**Table 1:** The primers of 10 selected hypoxia genes.

Gene name	Primer sequences	
	Forward Primer	Reverse Primer
GAPDH	GGAGCGAGATCCCTCCAAAAT	GGCTGTTGTCATACTTCTCATGG
PGK1	TGGACGTTAAAGGGAAGCGG	GCTCATAAGGACTACCGACTTGG
SLC2A5	GAGGCTGACGCTTGTGCTT	CCACGTTGTACCCATACTGGA
TPI1	CTCATCGGCACTCTGAACG	GCGAAGTCGATATAGGCAGTAGG
B4GALNT2	CACTGAACACCCTTGCTGATG	CAGCTTCCGGTCACTGGTAG
TPST2	AGTCCTCGGTCTACCTGTCG	GGCGTACATCACCTCGATGG
FBP1	CGCGCACCTCTATGGCATT	TTCTTCTGACACGAGAACACAC
KLF7	AGACATGCCTTGAATTGGAACG	GGGGTCTAAGCGACGGAAG
SDC4	GGACCTCCTAGAAGGCCGATA	AGGGCCGATCATGGAGTCTT
PKP1	TTTGCCGTCGGACCAAAAGAT	GAACCTCGATTGGAGTGGCTC

### Date of Acquisition

Gene expression profiles (fragments per kilobase million, FPKM normalized) and the corresponding clinical parameters of 572 primary LUAD patients and healthy people were downloaded from The Cancer Genome Atlas–Lung Adenocarcinoma (TCGA-LUAD) (https://portal.gdc.cancer.gov/) and were used as the training set. Datasets GSE13213 from the microarray datasets generated by Agilent-014850 Whole Human Genome Microarray 4x44K G4112F (Probe Name version) were downloaded from Gene Expression Omnibus (https://www.ncbi.nlm.nih.gov/geo/query/acc.cgi?acc) and was used as external independent validation set, including 117 LUAD patients. In addition, five datasets, namely, GSE72094, GSE30219, GSE31210, GSE37745, and GSE81089 from the same chip platform (Affymetrix Human Genome U133 Plus 2.0 Array) were integrated into a new cohort and were used as the other validation set, namely, GEO-II LUAD group, which contained a total of 904 I–IV LUAD patients (I, 557; II, 178; III, 99; IV, 70) meeting the criterion. Batch effects from the five independent datasets above were corrected by using the ComBat function (sva R package).

### Construction of Gene Signature by Integrating Four Machine Learning Algorithms

A total of 572 transcriptome data from TCGA were divided into 513 LUAD tumors group and 59 normal tissues group. The R package DESeq2 was applied to perform the differential expression analysis of hypoxia-related gene between tumors group and normal tissues group, followed by plotting the volcano plots for differentially expressed hypoxia-related gene using R package ggplot2. The hypoxia-related differentially expressed genes (DEGs) were defined as |log2 fold change| > 0.05, *p* < 0.05. Then, dimensionality reduction was further performed on differentially expressed hypoxia-related gene in LUAD tumors based on survival data by using the weighted random forest and sliding windows sequential forward feature selection (SWSFS) method, which was realized by R package ranger, a weighted version of random forest. The SWSFS method was used to identify the top important hypoxia-related DEGs by increasing DEGs one by one to the random forest model by the order of variable importance score (VIS). In the RF model, the ordinate (left) represents the out of bagging (OOB)’ error rate, which measured the performance of different gene combinations consisting of a specific number of hypoxia-related DEGs. In the RF model having the lowest error rate, the current hypoxia-related DEGs combination was screened out for further analysis.

To enhance the accuracy and reliability of the established HAGS, we make further screening of hypoxia-related DEGs by training the XGBoost model using the xgboost package in R language. The XGBoost model was used to analyze the contribution of each hypoxia-related DEGs to survival state in 513 LUAD tumors group; the top-ranked hypoxia-related DEGs with the VIS value of 0.01above were screened out for further analysis. After screening by two methods mentioned above, we used the intersection of RF model and the XGBoost model to identify candidate genes, followed by employing the support vector machine–recursive feature elimination (SVM–RFE) algorithms (19). SVM–RFE has been widely used to rank features and select the most significant features subset for classification. In this study, the hypoxia-related DEGs subset with the best accuracy for classifying survival status was chosen to be the HAGS by the mean of fivefold cross-validation in the SVM predictive model.

Finally, the HAGS subset screened by three models above was determined by Gaussian mixture model (GMM), which is a very feasible approach and has a good hierarchical agglomerative clustering performance (20). Logistic regression analysis was used to construct combined models of different gene sets combinations to predict survival status in LUAD patients. The area under the curves (AUCs) were calculated by constructing the receiver operating characteristic (ROC) curves to assess the predictive value of all logistic regression models. Then, the GMM was used to cluster gene sets according to the AUC values of all different gene sets combinations. The gene sets combinations with the highest AUC will be selected and determined as the final HAGS subset to establish HAGS. Ultimately, the risk score of HAGS was determined through the optimal parameter of logistic regression analysis and was calculated by the formula: $\text{risk score}=\int_{i}^{n}C_{i}G_{i}$ , where *C_i_* represents the coefficient of gene i, and *G_i_* is the normalized expression value of gene i.

### Validation of the HAGS

The risk score formula above was used to calculate risk scores for each LUAD patient. Then, the median score of the LUAD individuals in the training and external validation groups was used as a risk cutoff value to classify all LUAD individuals into the high- and low-risk groups. The survival status, hypoxia-associated gene expression, and overall survival (OS) time was compared between the two subgroups *via* Kaplan–Meier analysis, respectively. The gene expression levels were normalized by log transformation for each gene. Principal component analysis (PCA) was performed to observe the clustering conditions of LUAD individuals in different risk levels, visualized by the “scatterplot3d” R package.

### Gene Ontology and Kyoto Encyclopedia of Genes and Genomes Pathway Enrichment Analyses

Next, the co-expression genes of differential hypoxia-associated gene between high- and low-risk LUAD patients were chosen to perform Gene Ontology (GO) and Kyoto Encyclopedia of Genes and Genomes (KEGG) analyses, which was conducted by using the clusterProfiler package. Enrichment significance thresholds were set at *p* < 0.05 and false discovery rate (FDR) <0.05. GO analysis was used to map all DEGs to GO terms in the GO database (http://www.geneontology.org/) to analyze the main functions of the DEGs. The KEGG pathway database (www.genome.jp/kegg/) is a synthetic database, which was used to analyze the biochemical pathways of the DEGs of interest.

### Independent Prognostic Factors Analysis of Risk Score and Construction of a Nomogram Prediction Model

After the extraction of clinical information (age, grade, and stage) of LUAD patients in the TCGA and GSE 13213 cohort, univariate and multivariate prognostic analyses were used to demonstrate whether the risk score could be an independent prognostic factor. Based on the multivariate Cox regression analysis for risk score and other clinicopathological factors by the rms R package, a clinically adaptable nomogram prediction model was established to predict the survival probability of 513 LUAD individuals in 1, 3, 5, and 8 years from the TCGA group. Then, the calibration analysis and time-dependent ROC (tROC) curve were used to evaluate the prognostic value of nomogram for LUAD patients.

### Correlation Analyses Between HAGS and the Immune Activity

The single-sample gene set enrichment analysis (ssGSEA), an application and extension of Gene Set Enrichment Analysis (GSEA) algorithm, calculates separate enrichment scores for each pairing of a sample and gene set. To explore the relationship between the HAGS and the immune activity, we uploaded the gene expression matrix data of LUAD patients from TCGA. For 513 LUAD patients, the infiltration levels of 16 types of immune cells and the activity of 13 immune-related pathways were quantified using enrichment scores calculated by ssGSEA algorithm in R package gsva. Then, the Spearman correlation analyses were performed to evaluate the correlation between the levels of risk score and the infiltration levels of immune cells and immune-related pathways by R packages, “ggcor.” Similarly, the Spearman correlation of infiltration levels for different immune cells and immune-related pathways were also performed to analysis possible relationships between them.

In addition, based on expression profiling data retrieved from the TCGA database, the ssGSEA was used to quantify the 29 infiltrating immune cells types and immune-related pathways of 513 LUAD in the training set, which was divided into 256 high-risk score groups and 257 low-risk score groups based on the risk score. Then, statistical difference between the two groups was compared by the Wilcoxon test. The mutation status of TP53, KRAS, and epidermal growth factor receptor (EGFR), which was calculated by package “maftools,” was also displayed to gain insights into the tumor mutation burden between low- and high-risk groups stratified by the risk score. The clinical features (gender and survival) and TNM stage of patients between the two groups were also illustrated as an annotation.

### Analysis of the Tumor Mutation Status in the Low and High HAGS Risk Score Groups

The tumor mutational burden (TMB) is defined as the total number of somatic/acquired mutations per coding area of a tumor genome (Mut/Mb) (21) and calculated as the number of non-synonymous protein coding variants divided by the total sequenced genome length. To inquire about the association between the TMB and HAGS risk, we next compared the tumor mutation status between the low and high HAGS risk score groups. First, the RNA-seq data of 513 LUAD samples in the TCGA group was annotated by the annotation files (gencode.v22.annotation.gene.probeMap). Then, the mutational data of TCGA samples was identified and matched against the somatic point mutation database (Genomic Data Commons Data Portal, https://portal.gdc.cancer.gov/), which was used to check for the presence of mutation in large populations of control individuals. Significantly mutated genes (*p* < 0.05) between the low and high HAGS risk groups and the interaction effect of gene mutations were analyzed by maftools; only genes mutating more than 50 times in at least one group will be considered. The statistical significance test for the proportion of mutation was evaluated by one-sided z-test and two-sided Chi-square, and *p* < 0.05 was considered as significant.

### Correlation Analysis Between Hypoxia-Associated Gene Expression and Drug Sensitivity

The drug sensitivity data used in our study were downloaded from the CellMiner database (https://discover.nci.nih.gov/cellminer/home.do). The CellMiner database includes rapid access to and comparison of gene expression levels of 360 microRNAs, 22,379 genes, and 20,503 compounds incorporating 102 Food and Drug Administration (FDA)-approved drugs (22, 23). First, the gene expression and drug sensitivity data from the same sample were downloaded. Then, the drug sensitivity data were filtered after clinical trials verification and FDA standard certification. Eventually, we combined the 10 hypoxia-associated gene expressions with the retained drug sensitivity data to perform the Spearman correlation analysis. Higher Spearman Cor value indicates a stronger correlation.

### Chemotherapeutic Response Prediction

Based on the largest publicly available pharmacogenomics database [the Genomics of Drug Sensitivity in Cancer (GDSC), https://www.cancerrxgene.org/], we further predicted the chemotherapeutic response for each patient with high and low risk in the TCGA group to evaluate the value of hypoxia-derived signatures for LUAD treatment in the clinic. The half-maximal inhibitory concentration (IC50) of 28 antitumor drugs recommended by The American Joint Committee on Cancer (AJCC) guidelines for cancer treatment were calculated using the R package “pRRophetic,” which could simultaneously construct prediction models using transcriptome and drug sensitivity data derived from GDSC and apply it to the transcriptome information of 513 LUAD samples to generate predicted drug IC50s for each sample. Finally, the difference in the IC50s of 30 common antitumor drugs between the high- and low-risk groups was compared using the Wilcoxon signed-rank test. The prediction process was implemented by R package “pRRophetic” where the samples’ half-maximal inhibitory concentration (IC50) was estimated by ridge regression, and the prediction accuracy was evaluated by 10-fold cross-validation based on the GDSC training set (24).

### External Validation of Proteins and Transcription Levels of the HAGS

Human Protein Atlas antibody-based protein expression data are freely available online from the Human Protein Atlas (HPA) (www.proteinatlas.org) (25), a comprehensive database that provides the information on the tissue and cell distribution of 26,000 human proteins. The protein expressions of 10 hypoxia-associated genes (*TPST2*, *SDC4*, *KLF7*, *SLC2A5*, *TPI1*, *FBP1*, *B4GALNT2*, *PGK1*, *PKP1*, and *GAPDH*) in normal and LUAD tumor tissues were investigated based on the results of specific antibodies obtained from HPA. The human model diagrams illustrating the organ biodistribution of 10 genes in the human body were generated using gganatogram, an R package for modular visualization of anatograms and tissues based on ggplot2.

## Results

### Construction and Validation of HAGS by Integrating Four Machine Learning Algorithms

**Figure 2:**
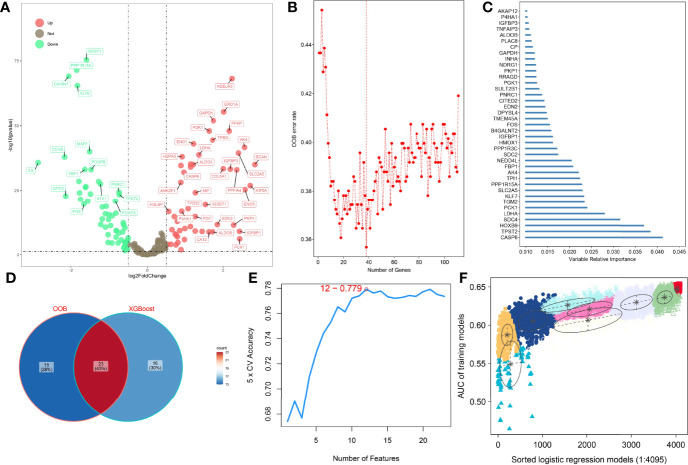
Four machine learning algorithms were integrated to establish the HAGS. **(A)** The DEGs between the tumors group and normal tissues group were illustrated with a volcano plot. **(B)** Supervised random forest models were used to identify the top important hypoxia-related DEGs. **(C)** Top 39 features selected using XGBoost and the corresponding variable importance score. x-Axis indicates the importance score, which is the relative number of a variable that is used to distribute the data; y-axis indicates the top 39 weighted variables. **(D)** The intersection of the random forest model and the XGBoost model. **(E)** The SVM–RFE algorithms were used to further screen gene set. **(F)** The GMM was used to determine the final HAGS.

To improve the reliability, validity, and accuracy of HAGS, we integrated four different machine learning algorithms to select the most reliable hypoxia-associated genes set. First, the hypoxia-related DEGs between tumors group and normal tissues group were illustrated with a volcano plot (**Figure 2A**), which was derived from “DESeq”-based differential gene expression analysis. Second, supervised random forest (RF) models were used to identify the top important hypoxia-related DEGs from the selected hypoxia-related DEGs (**Figure 2B**). Using RF–OOB algorithm, the subset of DEGs with the minimal value of OOB error was selected to be the optimal feature. Meanwhile, the top-ranked hypoxia-related DEGs were also generated by using the XGBoost algorithm based on the contribution (gain) of each hypoxia-related DEG to survival state (**Figure 2C**). Third, the intersection of the random forest model and the XGBoost model were analyzed by SVM–RFE algorithms to further screen gene set with the best accuracy for classifying survival status of LUAD patients (**Figures 2D, E**). Finally, from all different gene sets combinations selected through models above, the GMM was used to determine the final hypoxia-associated genes signature subset (**Figure 2F**), including *TPST2*, *SDC4*, *KLF7*, *SLC2A5*, *TPI1*, *FBP1*, *B4GALNT2*, *PGK1*, *PKP1*, and *GAPDH*. Based on the expression of these candidate genes, the risk score of HAGS for each patient in TCGA and GEO groups was calculated by the formula: $\text{Risk score}=\int_{i}^{n}C_{i}G_{i}$ , where Ci represents the coefficient of gene i, and Gi is the normalized expression value of gene i.

**Figure 3:**
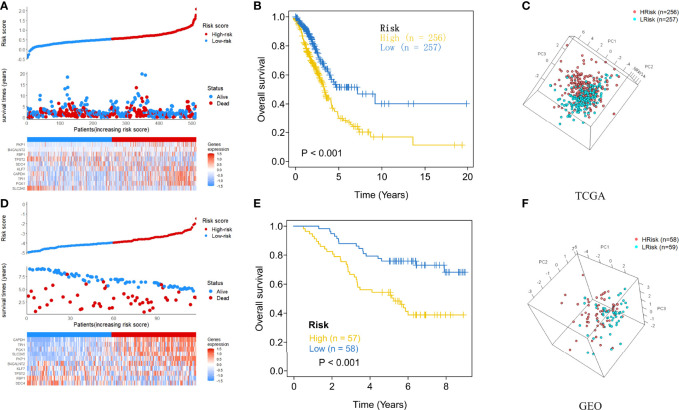
The risk score plots, OS status plots, and heatmaps of these 10 hypoxia-associated genes in the TCGA and GEO groups. **(A)** Risk score distribution, OS status, and the expression of 10 hypoxia-associated genes of LUAD patients in TCGA group. Red means high risk, blue means low risk. **(B)** Kaplan–Meier plot found that the HAGS divided patients into high- and low-risk groups with significant difference in OS. **(C)** Based on PCA analysis, the 513 LUAD patients in TCGA were distributed into two sections according to the risk score. **(D–F)** Similar results were also found in the GEO group. OS, overall survival; PCA, principal component analysis.

After each patient received a risk score according to the personalized formula of HAGS above, we divided patients in the TCGA training group into low‐risk (n = 257) and high-risk groups (n = 256) by using the median risk score as the threshold value. As show in **Figure 3A**, according to the median, all patients in the TCGA-LUAD group were distinctly stratified into low- and high-risk groups with the increasing risk score. By displaying the risk scores, survival status, and the expression of 10 hypoxia-associated genes in a dot plot or heat map, we found that patients with high-risk scores had higher expression of *PKP1*, *B4GALNT2*, *KLF7*, *GAPDH*, *TPI1*, and *PKP1*. Kaplan–Meier survival curves presented a significantly higher number of deaths in the high-risk group than in the low-risk group (*p* < 0.0001, **Figure 3B**), suggesting that the newly developed HAGS was able to effectively predict survival. Moreover, PCA analysis revealed that the individuals in different risk levels could be distinctly distributed into two sections based on the risk score (**Figure 3C**). Similarly, patients in GEO were also divided into low‐risk (n = 59) and high-risk groups (n = 58), and the results of Kaplan–Meier analysis and PCA were consistent with the results of the TCGA-LUAD group mentioned above (**Figures 3D–F**).

### Enrichment Analyses of GO and KEGG Pathways

**Figure 4:**
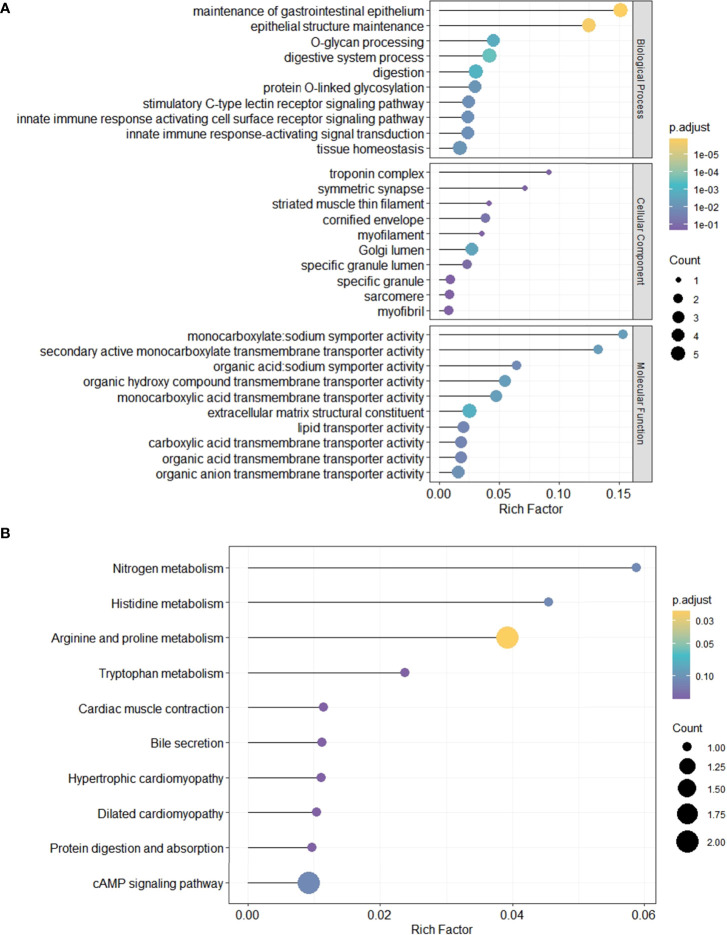
GO and KEGG pathway enrichment analyses of DEGs between high- and low-risk LUAD patients. *p*-value: purple, high (bottom); yellow, low (top). The size of the dots represents the number of DEGs. **(A)** GO analysis results. **(B)** KEGG pathway enrichment analyses results. *p*.adjust, adjusted *p*-value.

To evaluate the functional and biological implications of differentially expressed genes (DEGs) and further recognize important functional phenotypes of these genes between high- and low-risk LUAD patients, GO and KEGG pathways enrichment analyses of DEGs were performed, respectively. GO described DEGs in terms of their related biological processes, cellular components, and molecular function. Result from GO enrichment analyses illustrated that the DEGs were enriched in 30 GO terms, including 10 terms in biological processes, 10 terms in cellular component, and 10 terms in molecular function (**Figure 4A**). Additionally, 10 significant KEGG pathways were identified (**Figure 4B**), including one most significant types of pathways, namely, arginine and proline metabolism, proved to be an important metabolism pathway for lung cancer (26).

### Evaluation of Risk Score as an Independent Prognostic Factor for LUAD and Construction of a Nomogram for OS Prediction in LUAD Patients

**Figure 5:**
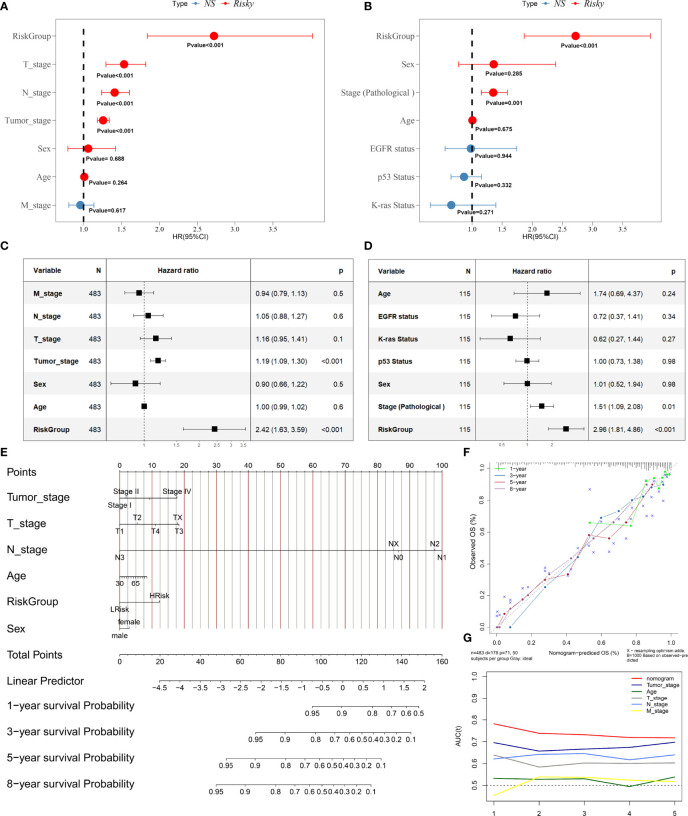
Evaluation of risk score as an independent prognostic factor and construction of nomogram for predicting overall survival in LUAD patients. **(A–D)** Results of the univariate and multivariate Cox regression analyses regarding OS in the TCGA and GEO. **(E)** Construction of the nomogram was based on sex, age, risk score, tumor stage, T stage, and N stage in the TCGA cohort. **(F)** Calibration plot analysis indicated that the nomogram showed a high accuracy of survival prediction. **(G)** tROC analysis demonstrated that the nomogram had the most powerful capacity for survival prediction by comparing with other clinicopathological factors.

After the extraction of clinical information (age, grade, and stage) of LUAD patients in the TCGA and GEO cohort, univariate and multivariate Cox regression analyses were performed to demonstrate whether the risk score derived from the HAGS model could serve as an independent prognostic factor for OS in LUAD patients. In the univariate Cox, the risk score was significantly associated with OS in both the training cohort from TCGA group and external validation dataset from GEO (*p* < 0.001, **Figures 5A, B**). The multivariate Cox regression analyses also indicated that the risk score was also proven to be an independent factor predicting OS in both TCGA and GEO cohorts (*p* < 0.001, **Figures 5C, D**).

Next, in order to acquire a more accurate quantitative method for disease progression and survival probability of LUAD patient, we constructed a nomogram to estimate the 1-, 3-, 5-, and 8-year survival probabilities of 513 patients with LUAD by integrating the risk score and different clinicopathological factors, including sex, age, risk score, tumor stage, T stage (tumor size), and N stage (lymph node metastasis) (**Figure 5E**). The calibration plots of the nomogram for 1-, 3-, 5-, and 8-year survival (**Figure 5F**) indicated that the OS estimated by the nomogram was extremely closely to the actual OS. Time-dependent ROC (tROC) curves of 5-year OS showed that the nomogram exhibited the most stable and powerful ability for predicting survival, with an average AUC above 0.7, much better than other clinicopathological factors (**Figure 5G**). These results further support the powerful discriminative ability of the HAGS in conjunction with clinicopathological factors for predicting survival in LUAD.

### Association Between HAGS Risk Score and the Clinical Characteristics of LUAD

**Figure 6:**
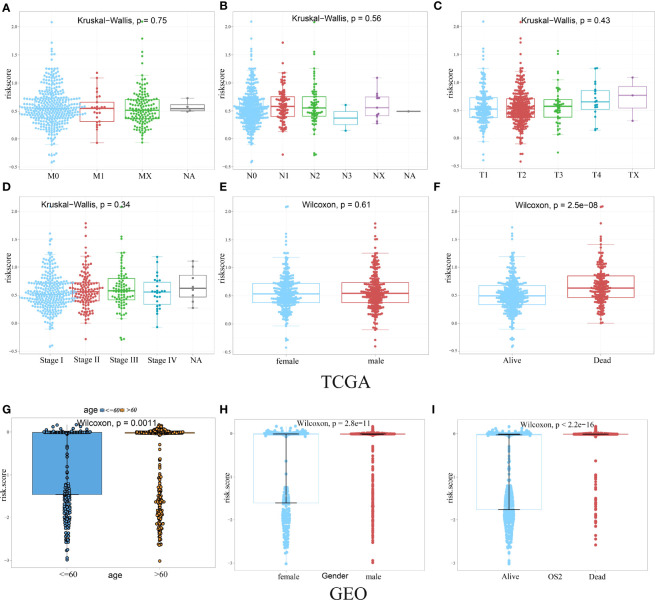
Difference analysis of the distribution of HAGS risk score in different TNM stage **(A–D)**, age **(G)**, gender **(E, H)**, and survival status **(F, I)**. Statistical difference of three or more groups was compared by the Kruskal–Wallis test and that of two groups was compared by the Wilcoxon test.

Given the diversity and complexity of different LUAD cases in clinical samples, we further investigated the distribution of the HAGS risk score in LUAD patients with different gender, age, survival status, and TNM stage. We found that there is no difference in LUAD patients with different TNM stage and gender in TCGA group (**Figures 6A–E**), only a significant difference was detected between patients with different survival status (*p* < 0.05, **Figure 6F**). In the GEO-II LUAD group, the elderly (>60), male, and dead populations all had significantly higher risk score than those in the younger (≤60), female, and alive populations, respectively (*p* < 0.05, **Figures 6G–I**).

**Figure 7:**
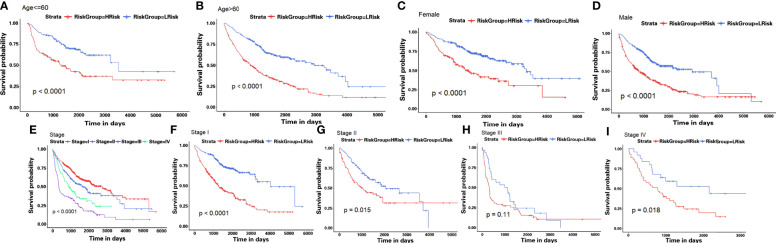
Kaplan–Meier survival analyses of the HAGS risk score in different subgroups. **(A–I)** LUAD patients in the low-risk group showed a more promising OS than the high-risk group in all subgroups (*p* < 0.001).

Then, in order to explore whether the 10-gene signature could be widely and accurately used to determine the survival conditions in different clinical characteristics, the Kaplan–Meier curves analysis was conducted in different subgroups with different age (≤60 and >60), gender (male and female), and stage (I–IV) from the GEO-II LUAD group. The results indicated that individuals in the low HAGS risk group had significantly better OS than individuals in the high HAGS risk group for all subgroups (*p* < 0.001, **Figures 7A–H**). These results also demonstrated that the hypoxia-associated signatures had reliable ability for predicting the OS of different subgroups, regardless of the difference in age, gender, and stage.

### Correlation of TMB With Hypoxia-Associated Signatures in LUAD

**Figure 8:**
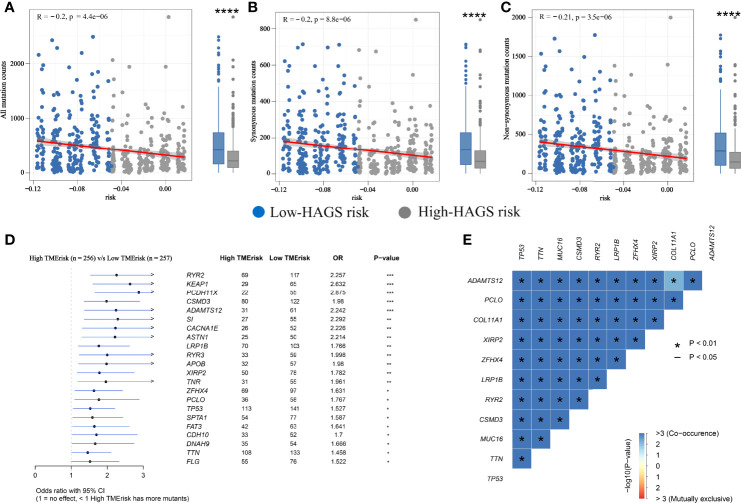
Hypoxia-associated signatures were related to TMB. **(A–C)** Association between all mutation counts, synonymous mutation counts, non-synonymous mutation counts, and HAGS risk score and their distribution in the low and the high HAGS risk groups. **(D)** Forest plot of genes mutating differentially between the low and the high HAGS risk groups. **(E)** Interaction effect of genes mutating differentially between the low and the high HAGS risk groups. *p < 0.05; **p < 0.01; ***p < 0.001; ****p < 0.0001.

We also checked for the somatic mutation in the Genomic Data Commons (GDC) data portal of the National Cancer Institute (https://portal.gdc.cancer.gov/) to investigate HAGS risk-related mechanisms based on TMB in LUAD. A comparison of cumulative mutant frequency between samples of the low- and the high-HAGS risk groups showed that less somatic mutations were observed in the high-HAGS risk group, including non-synonymous and synonymous mutations (**Figures 8A–C**). Concurrently, maftools analysis results showed that 22 mutated more frequently in LUAD patients in the low HAGS-risk group, including *RYR2*, *KEAP1*, *PCDH11X*, *CSMD3*, *ADAMTS12*, *SI*, *CACNA1E*, *ASTN1*, *LRP1B*, *RYR3*, *APOB*, *XIRP2*, *TNR*, *ZFHX4*, *PCLO*, *TP53*, *SPTA1*, *FAT3*, *CDH10*, *DNAH9*, *TTN*, and *FLG* (**Figure 8D**). Moreover, significant co-occurrences were observed among these mutated genes (**Figure 8E**).

### Relationship Between HAGS and the Immune Activity

**Figure 9:**
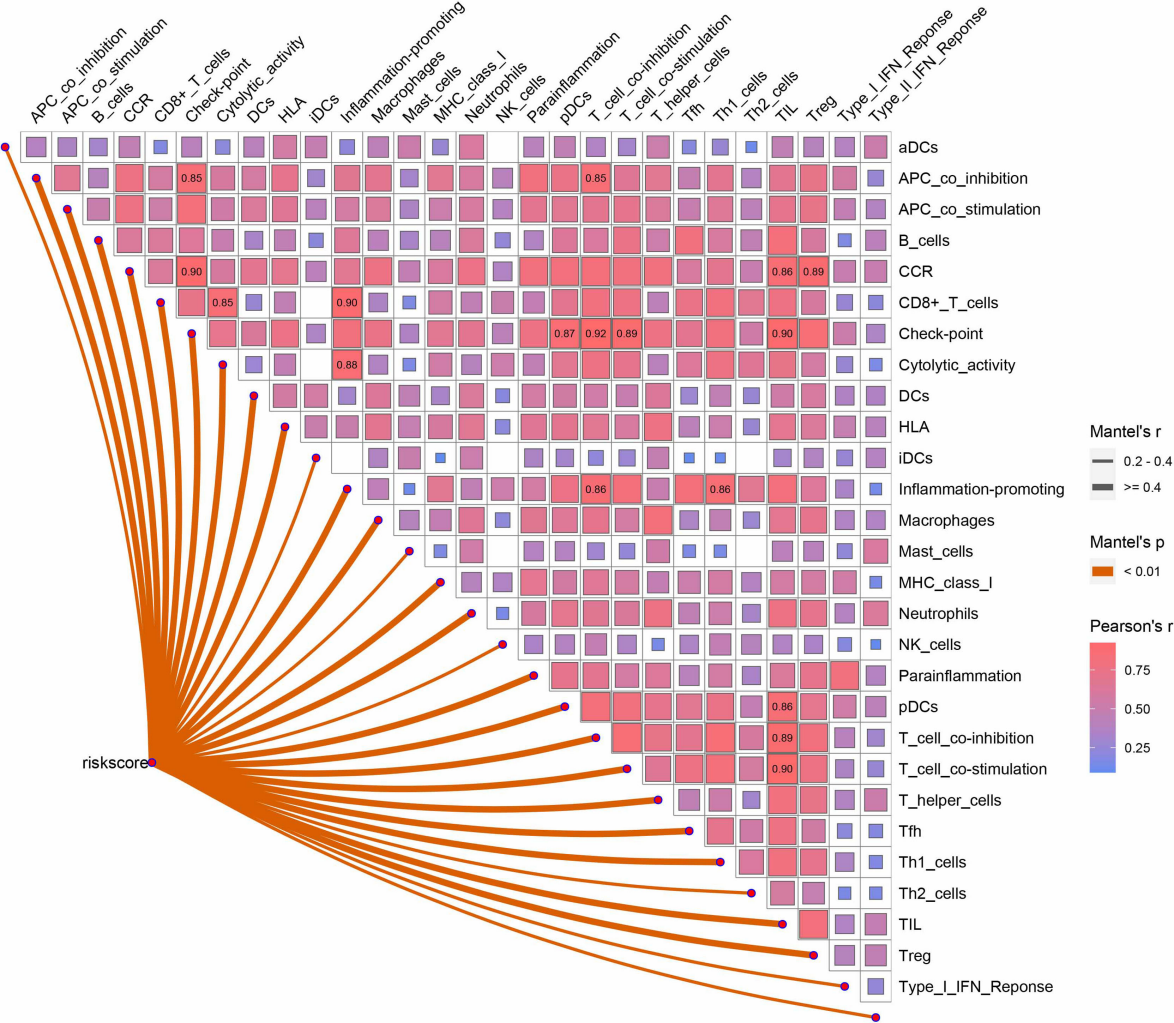
Correlation between HAGS and immune cells infiltration. The correlation between risk score and specific immune cells is shown with solid lines; the line color is related to the *p*-value. The square colors represent Pearson correlation coefficients between different immune cells; only the Pearson r ≥0.85 is displayed.

Correlation analyses between HAGS and the immune activity revealed that the risk scores in 513 LUAD patients were positively correlated with the levels of the APC co-inhibition, APC co-stimulation, B cells, CCR, CD8^+^ T cells, checkpoint, cytolytic activity, DCs, HLA, inflammation promotion, macrophages, major histocompatibility complex (MHC) class I, neutrophils, parainflammation, pDCs, T-cell co-inhibition, T-cell co-stimulation, T-helper cells, Tfh, Th1 cells, tumor-infiltrated lymphocyte (TIL), and Treg (*p* < 0.01, **Figure 9**). The Spearman correlation of different immune cells revealed that the expression levels of checkpoint was positively correlated with the levels of infiltrating CCR, T-cell co-inhibition, and TIL, respectively; the expression levels of inflammation promotion was positively correlated with the levels of CD8^+^ T cells; TIL was positively correlated with the T-cell co-stimulation (r ≥ 0.90). Those genes with strong correlations may also be functionally correlated, and future studies about hypoxia could incorporate them into existing knowledge.

**Figure 10:**
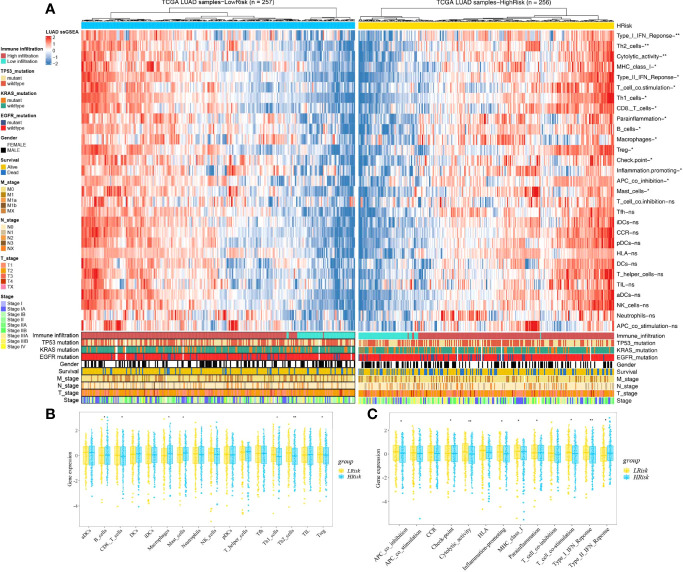
Landscape of immune cell infiltrations in the low and high TME risk groups. **(A)** The heatmap shows the normalized scores of immune cell infiltrations. Blue represents cells with lower infiltration, and red represents cells with higher infiltration. **(B, C)** The statistical difference between the two groups was compared by the Wilcoxon test. *p < 0.05; **p < 0.01; ns, not significant. In the lower panel, mutation status of TP53, KRAS, and EGFR; gender, survival; TNM stage; and stage were annotated.

Based on the ssGSEA, we further compared the enrichment scores of 16 types of immune cells and the activity of 13 immune-related pathways between the low- and high-risk groups in the TCGA cohorts. The comparison of the immune activity level between high- and low-risk groups in the TCGA dataset revealed that the high-risk subgroup generally showed lower activity of immune-related pathways and had lower levels of infiltration of immune cells, such as type I interferon (IFN) response, Th2 cells, cytolytic activity, MHC class I, T-cell co-stimulation, Th1 cells, CD8 T cells, parainflammation, Treg, checkpoint, inflammation promotion, and APC co-inhibition, than those in the low-risk group (**Figure 10**, *p* < 0.05), whereas only the levels of type II IFN response, B cells, macrophages, and mast cells in the high-risk group were significantly higher than those in the low-risk group, suggesting that the HAGS presented an excellent consistency with the immune activity.

### Drug Sensitivity Analysis of Hypoxia-Associated Gene

**Figure 11:**
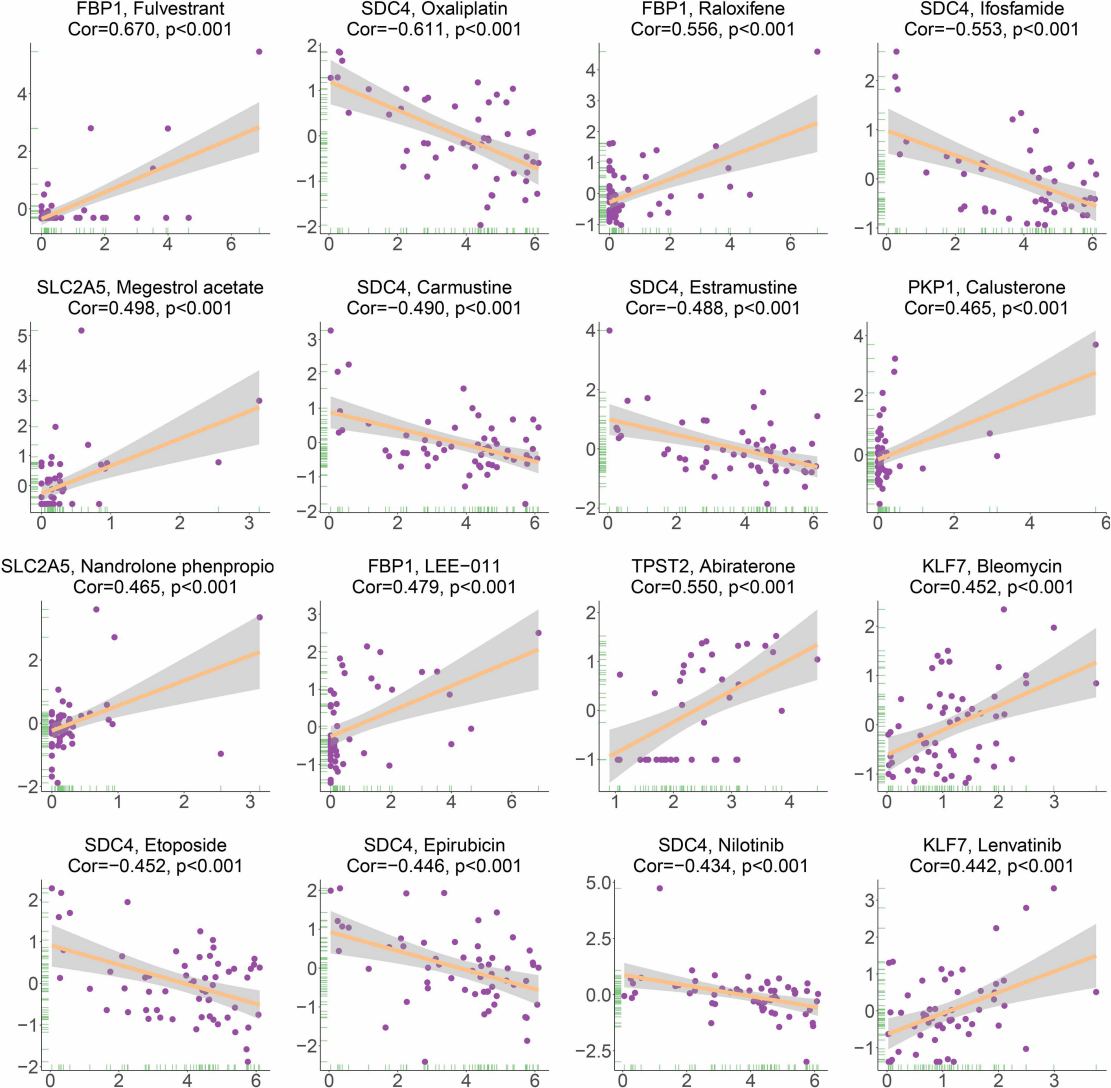
Correlation between HAGS and drug sensitivity analysis.

Based on the analysis of the correlation between 10 hypoxia-associated genes and drug sensitivity, significant correlation was found between the expression levels of the 10 genes and drug sensitivity (*p* < 0.001, **Figure 11**). The higher the expression of FBP1, the stronger the drug sensitivity of fulvestrant, raloxifene, and LEE-011 (*p* < 0.001). The higher the expression of SDC4, the weaker the drug sensitivity of oxaliplatin, ifosfamide, carmustine, estramustine, etoposide, epirubicin, and nilotinib (*p* < 0.001). SLC2A5 expression had a significant positive relationship with the drug sensitivity of megestrol acetate and nandrolone phenpropio (*p* < 0.001). The higher the expression of PKP1 and TPST2, the stronger the drug sensitivity of calusterone and abiraterone, respectively (*p* < 0.001). The expression of KLF7 had a significant positive relationship with the sensitivity of bleomycin and lenvatinib (*p* < 0.001).

### Comparison of the Sensitivity to Anticancer Drugs Between LUAD Patients With Different Hypoxia-Associated Risk Scores

**Figure 12:**
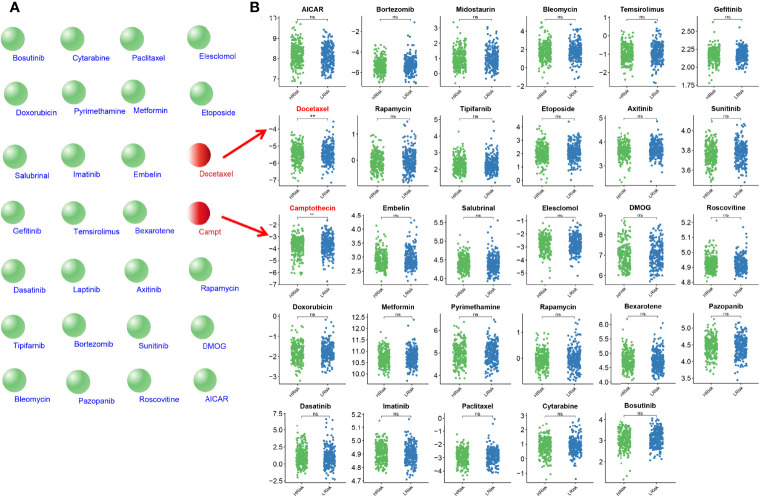
Estimated drug sensitivity in LUAD patients with high and low HAGS risk. **(A)** The 28 common anticancer drugs may have therapeutic potential for LUAD patient. **(B)** The difference in the IC50s of 30 common antitumor drugs between the high- and low-risk groups was compared by using the Wilcoxon signed-rank test. **p < 0.01; ns, not significant.

To further explore the value of hypoxia-associated gene sets for therapy in LUAD patient, we estimate the IC50s of the 28 common anticancer drugs for each sample through the expression matrix of hypoxia-associated gene in each LUAD sample from TCGA group. A comparison between the high- and low-risk groups found that the IC50s of docetaxel and camptothecin (Campt), two FDA-approved chemotherapeutics for cancer treatment, were higher in patients with lower HAGS risk score, which suggests that increased HAGS risk was accompanied by increased sensitivity to docetaxel and Campt (**Figure 12**). In other words, these two drugs may have the therapeutic potential to treat LUAD patient with HAGS high risk.

### Validation of the Expression of the HAGS

**Figure 13:**
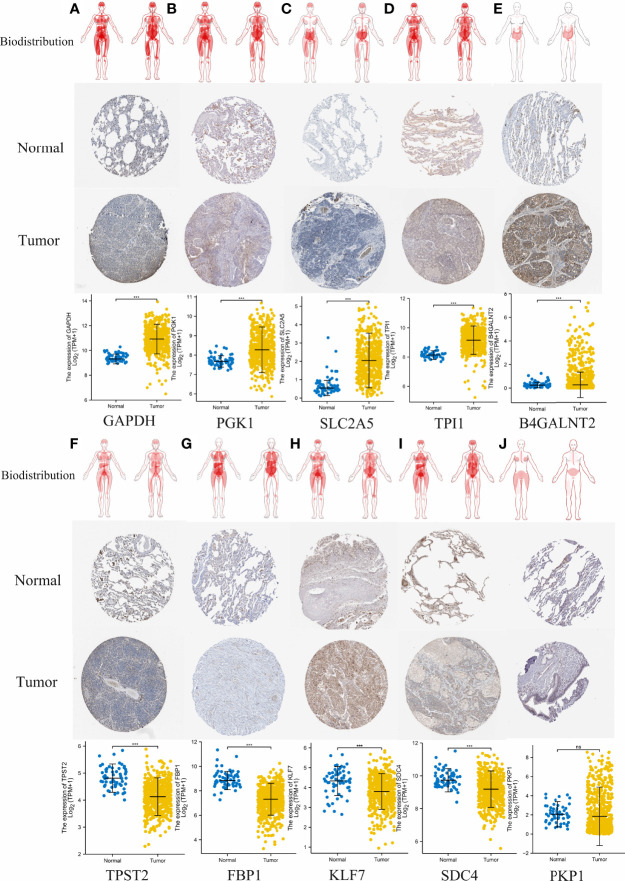
Comparison of hypoxia-associated gene expression at the protein level. From top to bottom, panels **(A–J)** represent biodistribution, IHC staining of protein expression in normal tissues and LUAD tumors tissues, and comparison of expression levels between normal tissues and LUAD tumors tissues for each gene, respectively. **(A)** GAPDH. **(B)** PGK1. **(C)** SLC2A5. **(D)** TPI1. **(E)** B4GALNT2. **(F)** TPST2. **(G)** FBP1, **(H)** KLF7, **(I)** SDC4. **(J)** PKP1. ***p < 0.001; ns, not significant, *p* > 0.05.

To evaluate differences in hypoxia-associated gene expression at the protein level, images of immunohistochemistry (IHC) staining of protein expression in normal tissues and LUAD tumors tissues were downloaded from the HPA and analyzed. As showcased in **Figure 13**, the protein expression level of five of these genes (*GAPDH*, *PGK1*, *SLC2A5*, *TPI1*, and *B4GALNT2*) was prominently higher in LUAD cancers when compared to the normal tissue (*p < 0.05, **Figure 13**). Otherwise, four of these genes (*TPST2*, *FBP1*, *KLF7*, and *SDC4*) were expressed at a low level, and no difference in expression levels of PKP1were noted between normal tissues and LUAD tumors tissues.

### Validation Experiment of Clinical Samples From LUAD Patients at the Gene Transcript Levels

**Figure 14:**
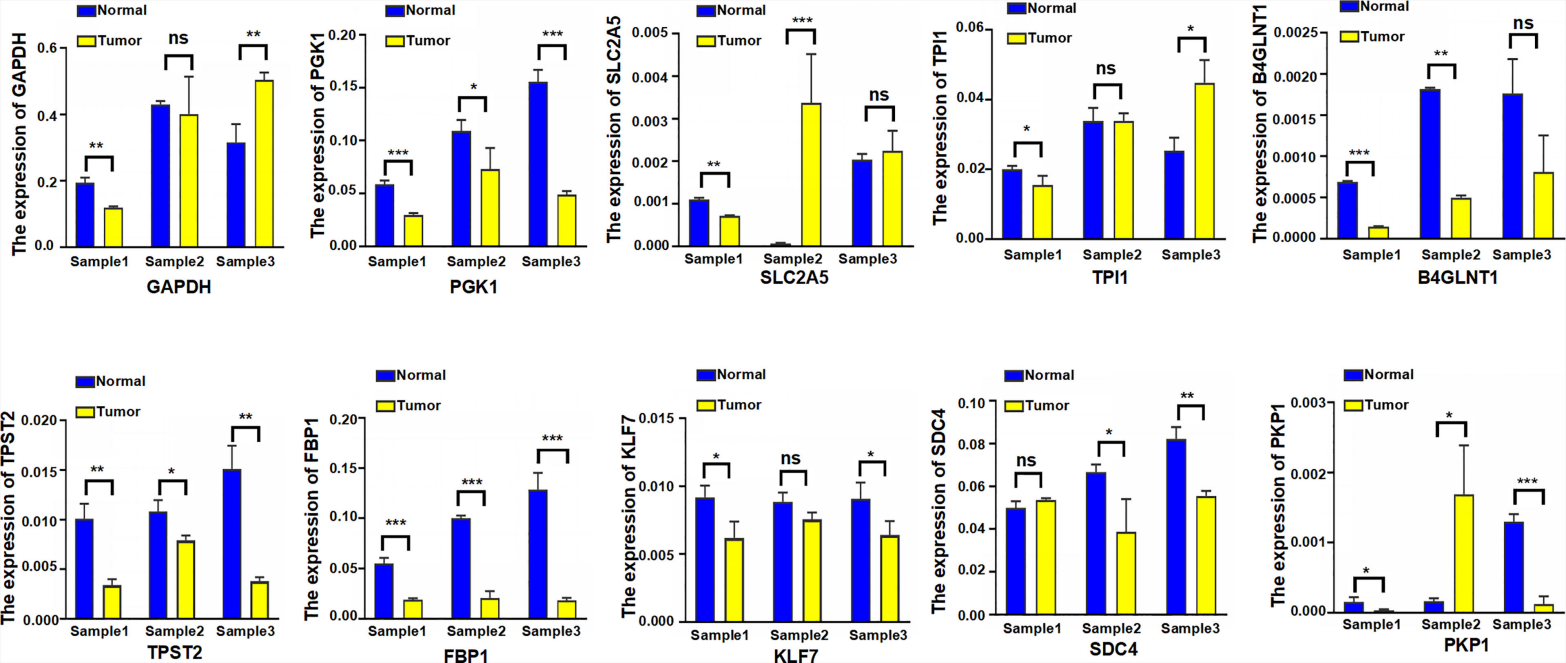
The expression level of 10 hypoxia-associated genes were verified at transcript levels by using three paired samples of LUAD tissues and the paired non-tumorous lung tissues. Sample1, Sample2, and Sample3 were collected from three different LUAD patients; each sample was used to detect 10 hypoxia-associated genes simultaneously. *p < 0.05; **p < 0.01; ***p < 0.001; ns, not significant.

Eventually, the expression level of 10 hypoxia-associated genes was verified at transcript levels. It is heartening to note that the expression level of all 10 hypoxia genes showed significant differences at least two paired samples of three LUAD tissues and the paired non-tumorous lung tissues (**Figure 14**).

## Discussion

As the most commonly diagnosed histological type of lung cancer, LUAD severely affects human health and possesses both extremely high morbidity and mortality in clinic (27). LUAD is the leading cause of cancer death worldwide, and its incidence is increasing worldwide (28). Notably, even at an early stage, LUAD patients also hold a high metastasis rate and present different prognosis (29). Studies investigating LUAD-associated genes may improve the prognosis, diagnosis, treatment, and prognosis assessment of LUAD patients. In the last few decades, a multitude of genes related to hypoxia have been identified and studied in various cancers (30–32). However, although numerous studies have explored the relationship between hypoxia and tumor formation, the deep-seated relationship between hypoxia-associated genes set and prognosis of LUAD patients remains quite limited.

In the present study, we developed a new HAGS (HAGS) by integrating four machine learning algorithms to predict clinical outcomes and therapeutic responses in LUAD patients, followed by performing internal and external validation for its performance in TCGA and GEO groups, respectively. Our results demonstrate that HAGS, as an independent prognostic factor, had a considerable effect on predicting the OS of LUAD patients. LUAD patients in the high HAGS risk group presented worse OS, lower TMB, and lower immune activity. Moreover, we revealed that the hypoxia-associated gene had a strong statistical association with the drug sensitivity of multiple FDA-approved drugs and had potential therapeutic value for LUAD patients based on the chemotherapeutic response prediction. Finally, validation studies on the expression levels of 10 hypoxia-associated genes were further analyzed to comprehensively confirm the reliability of selected gene set.

Unlike most previous studies that only use one single machine learning approach, our study established HAGS by integrating four different machine learning approaches to maximally improve the accuracy of our model. Finally, 10 hypoxia-associated genes (*TPST2*, *SDC4*, *KLF7*, *SLC2A5*, *TPI1*, *FBP1*, *B4GALNT2*, *PGK1*, *PKP1*, and *GAPDH*) were identified and combined as HAGS. Among these 10 hypoxia-associated genes, only *PGK1* and *GAPDH* are well-known hypoxia-regulated genes; the hypoxia-based function of *PGK1* and *GAPDH* have been adequately validated in lots of studies (33–36). Tyrosylprotein sulfotransferase 1 and 2 (*TPST-1* and *TPST-2*) are both responsible for the catalysis of tyrosine sulfation of chemokine receptors, such as *CXCR4* (Refs 93, 95, 96, 97, 98, 99) (37), it has previously been demonstrated that the *TPST 1* expression was significantly associated with lymph node metastasis and the TNM stage in patients with lung cancer and may be a negative prognostic biomarker of lung cancer (38, 39). However, the studies depicting the function of *TPST2* in cancer are extremely rare, so that the screen of this gene in our study indicates that its in-depth investigation in LUAD or other cancers should be performed to elucidate its underlying mechanisms. Sulfate proteoglycan syndecan-4 (*SDC4*) is an important member of Syndecans (SDCs) family, which is a family of transmembrane heparan sulfate proteoglycans (HSPGs) ubiquitously expressed on cell surfaces in mammals and plays a critical role in cell adhesion, migration, proliferation, differentiation, and angiogenesis through independent and growth factor mediated signaling (40). It has already been demonstrated that the *SDC4* exhibited multiple functions in tumor pathogenesis and progression (41), but the in-depth knowledge about *SDC4* is still very limited. For example, recently, Yang et al. for the first time identified *SDC4* as a direct anti-hepatocellular carcinoma (HCC) cellular target of bufalin in inhibiting cell proliferation, invasion, and angiogenesis (42). These indicated that the functional importance of *SDC4* in tumors, especially its roles in hypoxia, still needs more studies. Krüppel-like factor 7 (*KLF7*) is a member of the *KLF* family of zinc finger transcription factors and has antioncogenic functions in multiple cancer, such as human oral squamous cell carcinoma (OSCC) (43), glioma (44), gastric cancer (45), endometrial cancer (46), ovarian cancer (47), and non−small cell lung cancer (48). There is evidence that *KLF7* and hypoxia work together to influence cell apoptosis, but it is not yet fully understood how they will act together to affect tumor development and progression (49). *SLC2A5*, which promotes lung adenocarcinoma cell growth and metastasis by enhancing fructose utilization, was proven to be overexpressed in LUAD, and the expression was associated with prognosis (50). The result of IHC staining from the HPA also demonstrated that the protein expression of SLC2A5 was significantly overexpressed in LUAD tumors tissues compared to the normal tissues (**Figure 13C**). However, the regulation of *SLC2A5* in lung cancer has not been fully elucidated, especially when hypoxia is involved (50). *TPI1* (triosephosphate isomerase 1) was overexpressed in various types of cancers and might be induced by hypoxia in pan-cancer (51). *FBP1* (fructose-1,6-bisphosphatase) is known as a rate-limiting enzyme in gluconeogenesis, which is an important process in cell energy metabolism. The association between *FBP1* expression status and hypoxia had just been found in recent years, and relevant research is very limited (52). Tumor-hypoxia-related studies that are directly relevant to *B4GALNT2* and *PKP1* in hypoxia are few and far between.

Previous studies did not investigate these 10 hypoxia-related genes as a signature to predict the clinical outcomes of LUAD patients. A majority of these 10 hypoxia-related genes are involved in the complex regulation of progression in LUAD or other cancers. Considering the complexity of the genetic network, tumor progression is more likely to depend on the systematical interaction network based on a group of critical hypoxia-related genes rather than a single one. Therefore, the HAGS, that is, a comprehensive gene set combining 10 hypoxia-related genes, exhibited a powerful predictive prognostic capacity for LUAD patients. Univariate and multivariate Cox regression analyses both indicated that the HAGS was an independent prognostic factor in LUAD patients, more importantly, independently of age, gender, and stage (**Figure 7**). In addition, the independent and robust prognostic performance of HAGS was also confirmed by integrating the risk score and clinicopathological factors to construct a nomogram, which could be used to monitor the clinical outcomes of LUAD patients (**Figure 5**).

Recently, TMB is an emerging biomarker and has proved to be a potential and effective biomarker for independently predicting response to immunotherapy (53), but the effect and the prognostic role of the TMB on outcomes varied dramatically across cancer types (54, 55). Emerging pieces of evidence showed that higher TMB tends to form more new antigens, making tumors more immunogenic, improving clinical response to immunotherapy and prolonging the overall survival (56–58). This is in perfect agreement with our result that patients in the low-HAGS risk group showed more somatic mutations (**Figures 8A–C**), strong immune activity (**Figure 10**), and better OS (**Figures 3B, E** **)**. However, there were also studies showing the opposite, finding that high TMB was associated with worse prognosis (55, 59).

Recently, it was found that the drug responses and effect were influenced by hypoxia (60). Consistently, we found that the expression of certain hypoxia-associated genes had a significant positive relationship with the sensitivity of multiple drugs (**Figure 11**). In addition, we found a significant difference in IC50s of two anticancer drugs (docetaxel and camptothecin) between the high- and low-risk groups (**Figure 12**) by taking an integrative approach to analyzing the expression matrix of hypoxia-associated gene and the IC50s of the 28 common anticancer drugs in each LUAD sample. These signs suggested that hypoxia may exert a significant influence on drug sensitivity through the modulation of hypoxia-related pathways or genes, and more attention is require to study the effects of hypoxia on drug therapies.

## Conclusion

In summary, we developed a new hypoxia-associated gene signature (HAGS) by integrating four machine learning algorithms to predict clinical outcomes and therapeutic responses in LUAD patients, followed by performing internal and external validation for its performance in the TCGA and GEO groups, respectively. Our results demonstrate that HAGS, as an independent prognostic factor, had a considerable effect on predicting the OS of LUAD patients. LUAD patients in the high HAGS risk group presented worse OS, lower TMB, and lower immune activity. Moreover, we revealed that the hypoxia-associated gene had a strong statistical association with the drug sensitivity of multiple FDA-approved drugs and had potential therapeutic value for LUAD patients based on the chemotherapeutic response prediction. Finally, to comprehensively confirm the reliability of selected genes, validation studies on the expression levels of 10 hypoxia-associated genes were further analyzed at protein level and transcript levels.

## Data Availability Statement

The datasets presented in this study can be found in online repositories. The names of the repository/repositories and accession number(s) can be found in the article/supplementary material.

## Ethics Statement

The studies involving human participants were reviewed and approved by the Ethics Committee of the Ren Ji Hospital,affiliated Shanghai Jiao Tong University School of Medicine.

## Author Contributions

All authors listed have made a substantial, direct, and intellectual contribution to the work and approved it for publication.

## Funding

This work was supported by the innovative research team of high-level local universities in Shanghai, the National Natural Science Foundation of China (grant numbers 82102479, 81873957, and 81861138043), and the Shanghai Committee of Science and Technology, China (grant number 19JC1413005).

## Conflict of Interest

The authors declare that the research was conducted in the absence of any commercial or financial relationships that could be construed as a potential conflict of interest.

## Publisher’s Note

All claims expressed in this article are solely those of the authors and do not necessarily represent those of their affiliated organizations, or those of the publisher, the editors and the reviewers. Any product that may be evaluated in this article, or claim that may be made by its manufacturer, is not guaranteed or endorsed by the publisher.
